# Sensitization of supra-threshold pain responses—Translational aspects and mechanisms

**DOI:** 10.3389/fnetp.2022.1078890

**Published:** 2022-12-16

**Authors:** Robin Jonas, Martin Schmelz

**Affiliations:** ^1^ Department of Translational Pharmacology, Medical School EWL, Bielefeld University, Bielefeld, Germany; ^2^ UMCG Pain Center, Department of Anaesthesiology, University Medical Center Groningen, University of Groningen, Groningen, Netherlands; ^3^ Department of Experimental Pain Research, Medical Faculty Mannheim, University of Heidelberg, Mannheim, Germany

**Keywords:** ongoing pain, supra-threshold pain, short-term plasticity, negative results, dose-response relationship, glutamic acid, synaptic transmission, predictive validity

## Abstract

A substantial translational gap in pain research has been reflected by a mismatch of relevant primary pain assessment endpoints in preclinical vs. clinical trials. Since activity-dependent mechanisms may be neglected during reflexive tests, this may add as a confounding factor during preclinical pain assessment. In this perspective, we consider the evidence for a need for supra-threshold pain assessment in the pain research literature. In addition to that, we focus on previous results that may demonstrate an example mechanism, where the detection of neuron-glial interactions on pain seems to be substantially depending on the assessment of pain intensity beyond threshold levels.

## 1 The translational gap in pain research

The last 20 years has seen spectacular advances in our understanding of molecular mechanisms of pain. More than 200 relevant pain genes have been identified [e.g. Wistrom et al. (2022)], the molecular nature of transduction and transmission of sensory signals in primary nociceptors is being unraveled [e.g. Patapoutian et al. (2009)] and many of the neurotransmitters and receptors that modulate pain-related signals in the central nervous system are now known [e.g. Heinricher and Fields (2013)]. Several mutations affecting pain processing have been described, and there are large scale studies underway exploring the genetic variants that might have more subtle effects on pain processing and the epigenetic processes that might regulate them (Dib-Hajj et al., 2010; Bennett et al., 2019; Bali et al., 2021). While all of these mechanistic insights have been dramatic, remarkably little has changed with respect to the treatment of pain, at least in terms of available analgesic approaches, leaving a considerable proportion of chronic pain patients poorly treated (Jensen et al., 2001; Dahlhamer et al., 2018; Pitcher et al., 2019). Consequently, this perceived “failure” of animal studies for analgesic drug development has even raised the question if animal models are sufficient to predict analgesic efficacy in humans (Langley et al., 2008).

The translational gap in pain research may be partially explained by technical limitations to develop animal models that feature realistic approximations of human pathologies (Vierck et al., 2008; Mogil, 2009; Sadler et al., 2022). Unfortunately, these preclinical data may be necessary to find universally accepted objective biomarkers that are crucial to specifically define pathological subsets of pain, evaluate target engagement of new drugs and predict their analgesic efficacy (Davis et al., 2020). The predominant clinical problem of pain patients is suprathreshold, spontaneous pain, and many assays of suprathreshold pain in animals have been available and widely used for decades (e.g. the formalin test). The problem is they do not last very long. Since the focus in preclinical pain research has drifted to the ubiquitous study of “chronic” pain, researchers have been forced to revert to using evoked measures (Mogil and Crager, 2004; Sadler et al., 2022), because in the chronic assays often there are no spontaneous behaviors to measure (Langford et al., 2010; Gregory et al., 2013). On the contrary, it has been suggested that measuring pain thresholds could lack clinical relevance because it may not reflect the major clinical problem which is spontaneous or ongoing pain rather than evoked pain responses (Mogil et al., 2010; Bennett, 2012; Mogil, 2012). This may be particularly important for the development of novel analgesics for neuropathic pain, where a predominant focus on reflexive measures may be oversimplified and thereby potentially explains an apparent mismatch of primary pain assessment endpoints in preclinical vs. clinical trials (Fisher et al., 2020; Schmelz, 2020).

## 2 Short-term plasticity effects on pain

A broad spectrum of translational challenges such as difficulties in experimental designs [e.g. Tappe-Theodor et al. (2019)], species differences [e.g. Mogil (2019)] and potential modulation of evoked sensory reflexes by descending cognitive control [e.g. Wallwork et al. (2017); Dhondt et al. (2019)] have already been extensively discussed. However, despite all these remarkable arguments, another confounding factor which may come with the assessment of pain thresholds has been largely dismissed so far, and this is the temporal integration of axonal excitability or short-term plasticity and its resulting effects on pain. Considering that the general function of the withdrawal reflex is to avoid the pain evoking stimulus, corresponding neuronal activity is concomitantly terminated by the withdrawal action. Under these circumstances, the painful stimulus is potentially even terminated at a level when nociceptive firing rates may just reach clinical relevance. In turn, any mechanism which could be immediately affecting direct or indirect activity-dependent changes in neuronal excitability or synaptic transmission within the assessed nociceptive system will be neglected during this reflexive test. This effect could be particularly relevant for processes of short term plasticity which may be limiting trains of nociceptor activity (Tigerholm et al., 2014). As an example, these mechanisms may play a substantial role for the contrasting accommodation of pain upon prolonged electrical stimulation between healthy subjects and neuropathic pain patients (Jonas et al., 2018), or the sensitization of nociceptors after nerve growth factor (NGF) treatment (Schnakenberg et al., 2021). Interestingly, although both studies clearly demonstrate that the electrical stimulus is painful straight away from the beginning, differences in pain ratings between sensitized and non-sensitized areas have been shown to become only evident after prolonged (20 s) stimulation (Jonas et al., 2018; Schnakenberg et al., 2021). Therefore, albeit generally supporting the need of supra-threshold pain assessment, these studies also indicate that the level of pain perceived in response to a constant stimulation of C-nociceptors changes over time (1 min), and this effect consequently necessitates a prolonged assessment of ongoing pain to study mechanisms of activity dependent short-term plasticity. In turn, similar effects may also explain why quantitative sensory testing (QST) thresholds have been shown not to relate to clinical pain levels (Amiri et al., 2021; Forstenpointner et al., 2021) and therefore appear to provide no additional value for treatment or diagnosis of pain. It is important that Forstenpointner et al. published their results confirming the null hypothesis (Forstenpointner et al., 2021). However, more of resembling results are likely to be drawered (Finnerup et al., 2015), and publication of these data may not only reduce the translational gap in pain research, but also be helpful to overcome a perceived replicability crisis (Baker, 2016; Ventura, 2022).

## 3 Supra-threshold pain assessment for postoperative Pain

Taking into account that activity-dependent effects of short-term plasticity on pain are clinically relevant in pain patients, this mismatch may add to the array of confounding variables which are blurring the predictions of mechanistically driven translational approaches based on reflexive measures. This interference may become even more clear for the prediction of postoperative pain from preoperative pain assessments, where a substantial number of confounding variables, such as species differences or technical limitations in pain assessment, are even mitigated. Therefore, in analogy to predicted effects in translational approaches, the preoperative assessment of individual sensory phenotypes should guide predictions about the postoperative outcome. Correspondingly, low preoperative pain thresholds would be expected to indicate higher levels of postoperative pain and *vice versa*. However, predictive results in patients with postoperative pain are largely heterogeneous, with the most consistent predictive values for postoperative pain outcomes being supra-threshold pain assessments and dynamic parameters comprising temporal summation effects on pain (Abrishami et al., 2011; Sangesland et al., 2017; Petersen et al., 2021).

### 3.1 The spatiotemporal summation of glutamate

One significant advantage of supra-threshold and dynamic pain assessments is that they may reduce the translational gap in pain research by providing insights into potential mechanisms affecting ongoing nociceptive activity and short-term plasticity. Since discharge frequencies of primary afferent fibers and dorsal horn neurons positively correlate with the intensity of noxious stimuli, thereby encoding subjective levels of pain (LaMotte and Campbell, 1978; Handwerker et al., 1987; Cervero et al., 1988; Schmidt et al., 2000), relatively low discharge frequencies of primary afferent nociceptors are sufficient to provoke pain at threshold level (estimate of discharge frequency). At excitatory synapses, the level of released glutamate is characterized by a frequency-dependent increase that determines their transmission strength (Yamanaka et al., 1997; Carter and Regehr, 2000). Although this synaptic transmission is known to be ensured by rapid and spatially confined glutamate dynamics, there is increasing evidence that, under certain conditions, glutamate may escape from the synaptic cleft and accumulate in the extrasynaptic space (“glutamate spillover”) (Clements et al., 1992; Rothstein et al., 1996; Asztely et al., 1997). Such spatiotemporal summation of that neurotransmitter can lead to volume transmission and affect cooperative interactions between extrasynaptic high-affinity glutamate receptors or excitatory synapses (Barbour and Häusser, 1997; Arnth-Jensen et al., 2002; Okubo and Iino, 2011). This mechanism is of particular importance considering the physiological concept of “tripartite synapses” (Perea et al., 2009). It appears that especially the effect of volume transmission due to temporal summation is regulated by spinal glutamate transporters (Nie and Weng, 2009; Okubo and Iino, 2011). Even though it is suggested that glutamate transporters play a minor role in shaping the response to single stimuli (Isaacson and Nicoll, 1993), there is evidence that they limit the synaptic response to bursts of stimuli (Armbruster et al., 2016). These bursts can release glutamate at a level that may exceed the capacity of local clearance mechanisms, thereby allowing amplification of the local glutamate signal and even extrasynaptic glutamate spillover (Carter and Regehr, 2000). Therefore, based on higher levels of extrasynaptic glutamate, blocking of glutamate transporters could selectively amplify supra-threshold pain responses potentially without affecting withdrawal thresholds.

### 3.2 Glutamate spillover during supra-threshold nociceptive activity

**FIGURE 1:**
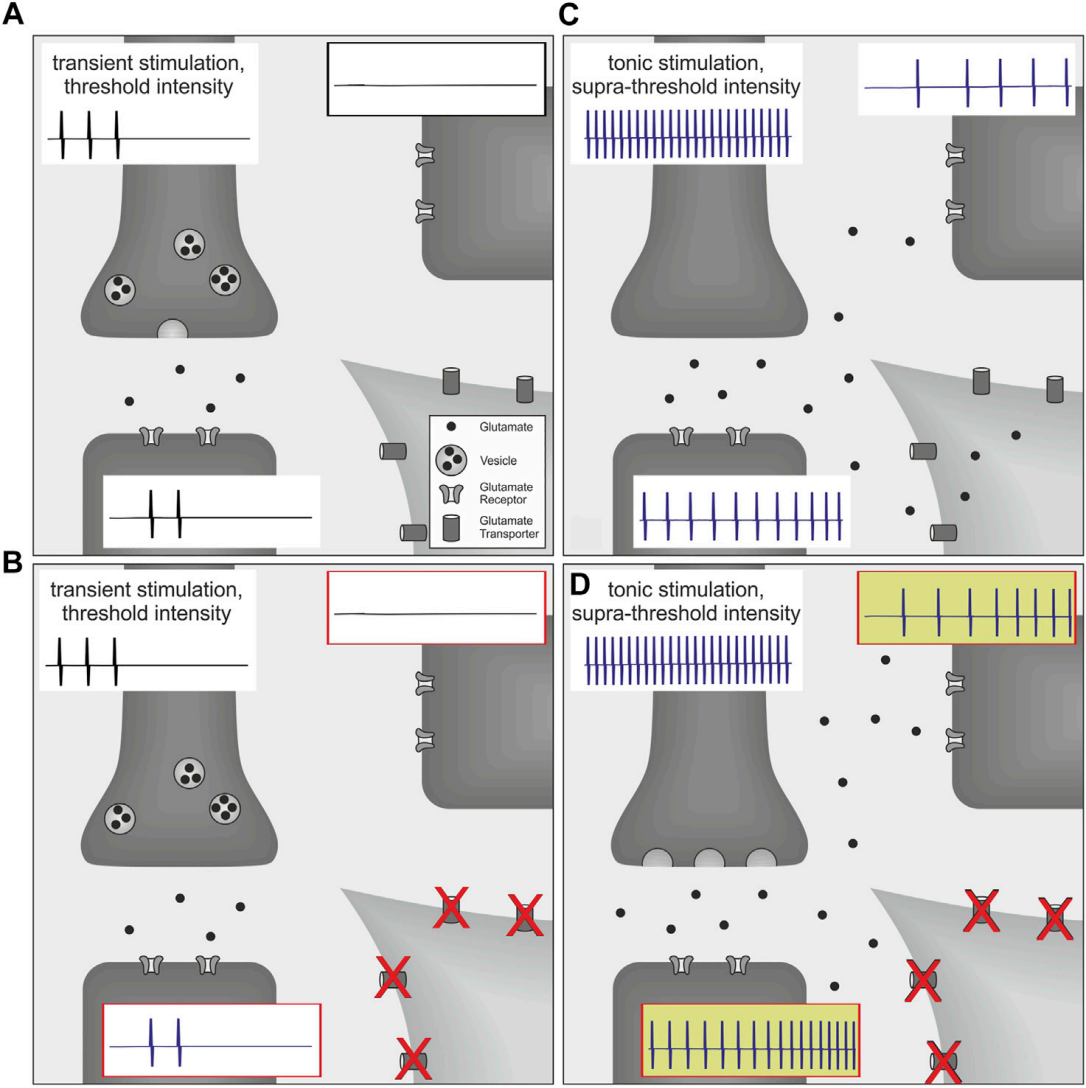
Schematic of volume transmission effects in the spinal cord on post-operative pain behavior. Each picture **(A–D)** illustrates synaptic transmission from the presynaptic terminal of a primary afferent nociceptor (dark gray, upper left; containing vesicles filled with glutamate) to postsynaptic terminals of two proximate neurons (dark gray, lower left and upper right; expressing glutamate receptors). Neuronal action potentials are indicated for each neuron by representative spikes (traces attached to respective cells). For simplicity, glutamate transporters are only illustrated on the perisynaptic glia cell (light gray, lower right). **(A)** At threshold intensity, discharge frequencies of nociceptors are considered to be low and neuronal firing will be substantially reduced by evoked withdrawal behavior. Therefore, glutamate release is low and spatiotemporal summation is expected to be limited by termination of the stimulus as well as initial clearance mechanisms such as neurotransmitter diffusion and reuptake. **(B)** Since these mechanisms are already sufficiently regulating synaptic glutamate concentration, blockage of glutamate transporters does not have any additional effect on synaptic transmission or secondary neuron activity. Therefore pain thresholds remain unchanged. **(C)** In contrast, a plantar incision causes tonic neuronal discharge which generates much higher levels of spatiotemporal glutamate accumulation, and this consequently increases activity of postsynaptic neurons. However, synaptic accumulation of glutamate also initiates additional clearing mechanisms *via* surrounding glutamate transporters that are countervailing glutamate spillover and volume transmission. **(D)** Under these circumstances, blockage of glutamate transporters directly reduces spinal glutamate clearance capacity and thereby increases non-evoked pain behavior.

Interestingly, in a rat model of postoperative pain, blocking spinal glutamate transporters with DL-threo-beta-benzyloxyaspartate (DL-TBOA) enhanced the level of ongoing pain behavior, whereas withdrawal thresholds to noxious heat or mechanical stimuli were not affected, even when higher doses were tested (Jonas, 2016). In this study, thermal and mechanical stimuli were directed to a restricted target area and applied with increasing intensity to behaviorally assess pain thresholds. At threshold intensity, withdrawal behavior is observed and the stimulus is terminated (Jonas, 2016). Discharge frequency of nociceptors at threshold intensity is expected to be low and consequently, glutamate release is low as decay of its concentration is fast based on diffusion and reuptake mechanisms (Clements et al., 1992; Rothstein et al., 1996; Asztely et al., 1997) (Figure 1A). Therefore, spatiotemporal summation of glutamate due to a massive release of this neurotransmitter is unlikely to occur when the noxious stimulus is terminated at threshold intensity (Figure 1B). In contrast, a plantar incision causes local inflammation as well as a possible additional neuronal damage. Activation and sensitization of nociceptors by inflammatory mediators (Koppert et al., 2004; Banik et al., 2005; Rukwied et al., 2010a,b; Schnakenberg et al., 2021) facilitate tonic neuronal discharge which might be sufficient to generate spatiotemporal summation of glutamate and consecutively lead to glutamate spillover and volume transmission (Carter and Regehr, 2000; Okubo and Iino, 2011). Under these circumstances, the amount of released glutamate has already exceeded the level which is required to evoke respective withdrawal responses. Therefore, increasing neuronal discharge by testing the animal with external stimuli will add to the spatiotemporal summation of glutamate, and this effect seems to be unlikely to be reflected by corresponding changes of pain thresholds. On the other hand, supra-threshold stimulation of the injury site and tonic nociceptor discharge from the inflamed tissue is expected to modify behavior as assessed by the non-evoked pain (NEP)-score. Higher scores are expected to be linked to higher levels of spinal glutamate and possibly associated with the accumulation of glutamate in the extrasynaptic space (Figure 1C). In analogy to an increase of presynaptic glutamate release by additional stimulation of spontaneously discharging nociceptors, spatiotemporal summation of this neurotransmitter may also be increased by reduced clearance mechanisms. Since local glutamate transporters could be particularly important to limit glutamate accumulation, blocking of these transporters may augment glutamate spillover and consequently facilitate firing activity in postsynaptic neurons (Nie and Weng, 2009, 2010; Nie et al., 2010) (Figure 1D).

This may be particularly important for the clinical situation of postoperative patients, which is characterized by reduced pain thresholds and ongoing pain (Wilder-Smith and Arendt-Nielsen, 2006). However, suffering is linked to supra-threshold nociceptor activation, both in spontaneous or induced pain. Therefore, glutamate spillover and volume transmission could be important processes during supra-threshold nociceptive activity in ongoing and induced pain responses from postoperative pain patients. Indeed, upregulated expression of glutamate transporters by ceftriaxone has been shown to reduce pain in animals (Hu et al., 2010; Yang et al., 2011; Luo et al., 2020) and humans (Macaluso et al., 2013), suggesting that these transporters are highly relevant under conditions of strong nociceptor activation during postoperative pain states.

Moreover, there is clear evidence for a much broader role of this mechanism as spinal excitatory amino acid transporters (EAATs) contribute to experimental neuropathic (Temmermand et al., 2022), but also inflammatory pain (Zhang et al., 2021). Accordingly, a number of positive modulators of the glutamate transport system have shown analgesic effects in neuropathic and inflammatory pain conditions (Gegelashvili and Bjerrum, 2019).

It is interesting to note that suprathreshold and tonic nociceptors activation is not only linked to the induction of pain, but is also required for the induction of the “conditioning pain modulation (CPM)” representing a descending pain control mechanism. Pain levels for the induction of this descending pain control have been suggested as tonic and supra-threshold reaching 20 to 40 on a scale from 0 to 100 (Yarnitsky et al., 2015). Teleologically, such an arrangement might guarantee that descending analgesic effects are restricted to situations of “real need”, i.e. intense or ongoing pain conditions. Under these circumstances, volume transmission could increase central excitability by unmasking normally silent connections between spinal neurons and threreby facilitate segmental inhibition (Arendt-Nielsen and Gotliebsen, 1992; Valeriani et al., 2005). Accordingly, this mechanism may explain segmental effects of tonic CPM generating homotopic hypoalgesia without concomitant cortical electroencephalography (EEG) changes, whereas heterotopic stimuli are causing short-term cortical plasticity effects which are correlating to supra-threshold pain ratings (Egsgaard et al., 2012).

## 4 Conclusion

Taken together, this perspective supports the idea of using differential behavioral read-outs to assess several clinically relevant aspects of pain, i. e. spontaneous pain, lowered pain thresholds and increased supra-threshold encoding. Thus, we not only confirm that even negative results of reflexive tests may be complementing the holistic clinical picture with valuable insights into contributing nociceptive signaling mechanisms (Bordeleau et al., 2021; Vollert et al., 2021), but also accentuate that the assessment of ongoing nociceptive activity and supra-threshold pain responses are emerging as substantial tools to study translational aspects and mechanisms of pain (Mogil and Crager, 2004; Mogil, 2012; Schmelz, 2020, 2022).

## Abbreviations

EAAT, excitatory amino acid transporter; EEG, electroencephalography; CPM, conditioning pain modulation; NEP, non-evoked pain; NGF, nerve growth factor; DL-TBOA, DL-threo-beta-benzyloxyaspartate; QST, quantitative sensory testing.
